# COVID-19’s impacts on the scope, effectiveness, and interaction characteristics of online learning: A social network analysis

**DOI:** 10.1371/journal.pone.0273016

**Published:** 2022-08-23

**Authors:** Junyi Zhang, Yigang Ding, Xinru Yang, Jinping Zhong, XinXin Qiu, Zhishan Zou, Yujie Xu, Xiunan Jin, Xiaomin Wu, Jingxiu Huang, Yunxiang Zheng

**Affiliations:** 1 School of Educational Information Technology, South China Normal University, Guangzhou, Guangdong, China; 2 Hangzhou Zhongce Vocational School Qiantang, Hangzhou, Zhejiang, China; 3 Faculty of Education, Shenzhen University, Shenzhen, Guangdong, China; Central China Normal University, CHINA

## Abstract

The COVID-19 outbreak brought online learning to the forefront of education. Scholars have conducted many studies on online learning during the pandemic, but only a few have performed quantitative comparative analyses of students’ online learning behavior before and after the outbreak. We collected review data from China’s massive open online course platform called icourse.163 and performed social network analysis on 15 courses to explore courses’ interaction characteristics before, during, and after the COVID-19 pan-demic. Specifically, we focused on the following aspects: (1) variations in the scale of online learning amid COVID-19; (2a) the characteristics of online learning interaction during the pandemic; (2b) the characteristics of online learning interaction after the pandemic; and (3) differences in the interaction characteristics of social science courses and natural science courses. Results revealed that only a small number of courses witnessed an uptick in online interaction, suggesting that the pandemic’s role in promoting the scale of courses was not significant. During the pandemic, online learning interaction became more frequent among course network members whose interaction scale increased. After the pandemic, although the scale of interaction declined, online learning interaction became more effective. The scale and level of interaction in Electrodynamics (a natural science course) and Economics (a social science course) both rose during the pan-demic. However, long after the pandemic, the Economics course sustained online interaction whereas interaction in the Electrodynamics course steadily declined. This discrepancy could be due to the unique characteristics of natural science courses and social science courses.

## 1. Introduction

The development of the mobile internet has spurred rapid advances in online learning, offering novel prospects for teaching and learning and a learning experience completely different from traditional instruction. Online learning harnesses the advantages of network technology and multimedia technology to transcend the boundaries of conventional education [1]. Online courses have become a popular learning mode owing to their flexibility and openness. During online learning, teachers and students are in different physical locations but interact in multiple ways (e.g., via online forum discussions and asynchronous group discussions). An analysis of online learning therefore calls for attention to students’ participation. Alqurashi [2] defined interaction in online learning as the process of constructing meaningful information and thought exchanges between more than two people; such interaction typically occurs between teachers and learners, learners and learners, and the course content and learners.

Massive open online courses (MOOCs), a 21st-century teaching mode, have greatly influenced global education. Data released by China’s Ministry of Education in 2020 show that the country ranks first globally in the number and scale of higher education MOOCs. The COVID-19 outbreak has further propelled this learning mode, with universities being urged to leverage MOOCs and other online resource platforms to respond to government’s “School’s Out, But Class’s On” policy [3]. Besides MOOCs, to reduce in-person gatherings and curb the spread of COVID-19, various online learning methods have since become ubiquitous [4]. Though Lederman asserted that the COVID-19 outbreak has positioned online learning technologies as the best way for teachers and students to obtain satisfactory learning experiences [5], it remains unclear whether the COVID-19 pandemic has encouraged interaction in online learning, as interactions between students and others play key roles in academic performance and largely determine the quality of learning experiences [6]. Similarly, it is also unclear what impact the COVID-19 pandemic has had on the scale of online learning.

Social constructivism paints learning as a social phenomenon. As such, analyzing the social structures or patterns that emerge during the learning process can shed light on learning-based interaction [7]. Social network analysis helps to explain how a social network, rooted in interactions between learners and their peers, guides individuals’ behavior, emotions, and outcomes. This analytical approach is especially useful for evaluating interactive relationships between network members [8]. Mohammed cited social network analysis (SNA) as a method that can provide timely information about students, learning communities and interactive networks. SNA has been applied in numerous fields, including education, to identify the number and characteristics of interelement relationships. For example, Lee et al. also used SNA to explore the effects of blogs on peer relationships [7]. Therefore, adopting SNA to examine interactions in online learning communities during the COVID-19 pandemic can uncover potential issues with this online learning model.

Taking China’s icourse.163 MOOC platform as an example, we chose 15 courses with a large number of participants for SNA, focusing on learners’ interaction characteristics before, during, and after the COVID-19 outbreak. We visually assessed changes in the scale of network interaction before, during, and after the outbreak along with the characteristics of interaction in Gephi. Examining students’ interactions in different courses revealed distinct interactive network characteristics, the pandemic’s impact on online courses, and relevant suggestions. Findings are expected to promote effective interaction and deep learning among students in addition to serving as a reference for the development of other online learning communities.

## 2. Literature review and research questions

Interaction is deemed as central to the educational experience and is a major focus of research on online learning. Moore began to study the problem of interaction in distance education as early as 1989. He defined three core types of interaction: student–teacher, student–content, and student–student [9]. Lear et al. [10] described an interactivity/ community-process model of distance education: they specifically discussed the relationships between interactivity, community awareness, and engaging learners and found interactivity and community awareness to be correlated with learner engagement. Zulfikar et al. [11] suggested that discussions initiated by the students encourage more students’ engagement than discussions initiated by the instructors. It is most important to afford learners opportunities to interact purposefully with teachers, and improving the quality of learner interaction is crucial to fostering profound learning [12]. Interaction is an important way for learners to communicate and share information, and a key factor in the quality of online learning [13].

Timely feedback is the main component of online learning interaction. Woo and Reeves discovered that students often become frustrated when they fail to receive prompt feedback [14]. Shelley et al. conducted a three-year study of graduate and undergraduate students’ satisfaction with online learning at universities and found that interaction with educators and students is the main factor affecting satisfaction [15]. Teachers therefore need to provide students with scoring justification, support, and constructive criticism during online learning. Some researchers examined online learning during the COVID-19 pandemic. They found that most students preferred face-to-face learning rather than online learning due to obstacles faced online, such as a lack of motivation, limited teacher-student interaction, and a sense of isolation when learning in different times and spaces [16, 17]. However, it can be reduced by enhancing the online interaction between teachers and students [18].

Research showed that interactions contributed to maintaining students’ motivation to continue learning [19]. Baber argued that interaction played a key role in students’ academic performance and influenced the quality of the online learning experience [20]. Hodges et al. maintained that well-designed online instruction can lead to unique teaching experiences [21]. Banna et al. mentioned that using discussion boards, chat sessions, blogs, wikis, and other tools could promote student interaction and improve participation in online courses [22]. During the COVID-19 pandemic, Mahmood proposed a series of teaching strategies suitable for distance learning to improve its effectiveness [23]. Lapitan et al. devised an online strategy to ease the transition from traditional face-to-face instruction to online learning [24]. The preceding discussion suggests that online learning goes beyond simply providing learning resources; teachers should ideally design real-life activities to give learners more opportunities to participate.

As mentioned, COVID-19 has driven many scholars to explore the online learning environment. However, most have ignored the uniqueness of online learning during this time and have rarely compared pre- and post-pandemic online learning interaction. Taking China’s icourse.163 MOOC platform as an example, we chose 15 courses with a large number of participants for SNA, centering on student interaction before and after the pandemic. Gephi was used to visually analyze changes in the scale and characteristics of network interaction. The following questions were of particular interest:

(1) Can the COVID-19 pandemic promote the expansion of online learning?
(2a) What are the characteristics of online learning interaction during the pandemic?
(2b) What are the characteristics of online learning interaction after the pandemic?
(3) How do interaction characteristics differ between social science courses and natural science courses?

## 3. Methodology

### 3.1 Research context

We selected several courses with a large number of participants and extensive online interaction among hundreds of courses on the icourse.163 MOOC platform. These courses had been offered on the platform for at least three semesters, covering three periods (i.e., before, during, and after the COVID-19 outbreak). To eliminate the effects of shifts in irrelevant variables (e.g., course teaching activities), we chose several courses with similar teaching activities and compared them on multiple dimensions. All course content was taught online. The teachers of each course posted discussion threads related to learning topics; students were expected to reply via comments. Learners could exchange ideas freely in their responses in addition to asking questions and sharing their learning experiences. Teachers could answer students’ questions as well. Conversations in the comment area could partly compensate for a relative absence of online classroom interaction. Teacher–student interaction is conducive to the formation of a social network structure and enabled us to examine teachers’ and students’ learning behavior through SNA. The comment areas in these courses were intended for learners to construct knowledge via reciprocal communication. Meanwhile, by answering students’ questions, teachers could encourage them to reflect on their learning progress. These courses’ successive terms also spanned several phases of COVID-19, allowing us to ascertain the pandemic’s impact on online learning.

### 3.2 Data collection and preprocessing

To avoid interference from invalid or unclear data, the following criteria were applied to select representative courses: (1) generality (i.e., public courses and professional courses were chosen from different schools across China); (2) time validity (i.e., courses were held before during, and after the pandemic); and (3) notability (i.e., each course had at least 2,000 participants). We ultimately chose 15 courses across the social sciences and natural sciences (see Table 1). The coding is used to represent the course name.

**Table 1:** List of the target courses.

Course type	Course name	Coding
**Social science courses**	Educational Wisdom from the Analects of Confucius	SS1
	Personality Psychology	SS2
	The World’s Three Major Religions and Arts	SS3
	Legal Methodology	SS4
	Economics	SS5
	Principles and Methods of Instructional Design	SS6
	Approaching Marx	SS7
**Natural science courses**	Electrodynamics	NS1
	Java Language Programming	NS2
	College Physics I	NS3
	Advanced Mathematics I	NS4
	Machine Learning	NS5
	Pharmaceutical Chemistry	NS6
	Medical Statistics	NS7
	Cell Biology	NS8

**Fig 1:**
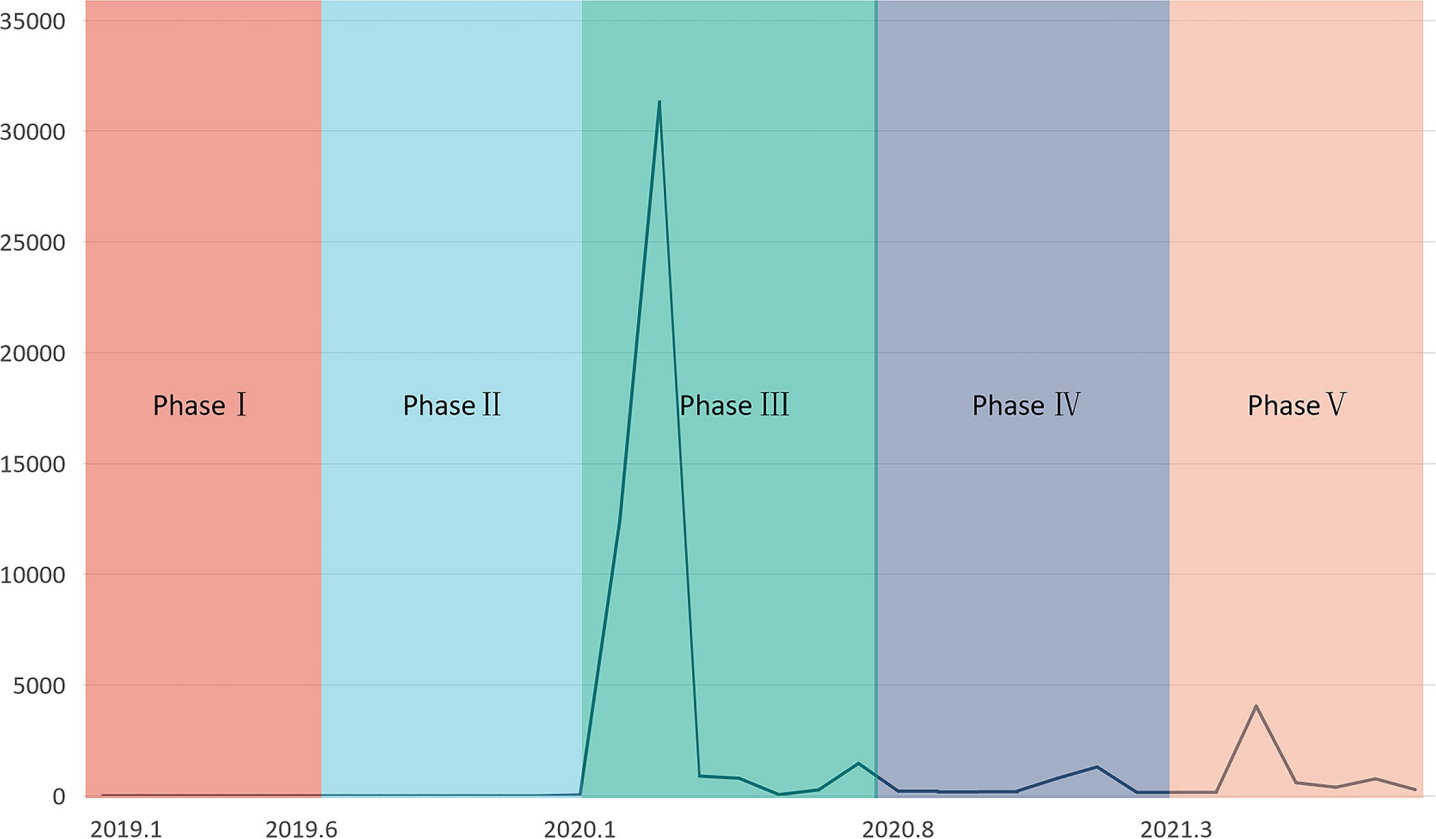
Number of confirmed cases in China.

To discern courses’ evolution during the pandemic, we gathered data on three terms before, during, and after the COVID-19 outbreak in addition to obtaining data from two terms completed well before the pandemic and long after. Our final dataset comprised five sets of interactive data. Finally, we collected about 120,000 comments for SNA. Because each course had a different start time—in line with fluctuations in the number of confirmed COVID-19 cases in China and the opening dates of most colleges and universities—we divided our sample into five phases: well before the pandemic (Phase I); before the pandemic (Phase Ⅱ); during the pandemic (Phase Ⅲ); after the pandemic (Phase Ⅳ); and long after the pandemic (Phase Ⅴ). We sought to preserve consistent time spans to balance the amount of data in each period (Fig 1).

### 3.3 Instrumentation

Participants’ comments and “thumbs-up” behavior data were converted into a network structure and compared using social network analysis (SNA). Network analysis, according to M’Chirgui, is an effective tool for clarifying network relationships by employing sophisticated techniques [25]. Specifically, SNA can help explain the underlying relationships among team members and provide a better understanding of their internal processes. Yang and Tang used SNA to discuss the relationship between team structure and team performance [26]. Golbeck argued that SNA could improve the understanding of students’ learning processes and reveal learners’ and teachers’ role dynamics [27].

To analyze Question (1), the number of nodes and diameter in the generated network were deemed as indicators of changes in network size. Social networks are typically represented as graphs with nodes and degrees, and node count indicates the sample size [15]. Wellman et al. proposed that the larger the network scale, the greater the number of network members providing emotional support, goods, services, and companionship [28]. Jan’s study measured the network size by counting the nodes which represented students, lecturers, and tutors [29]. Similarly, network nodes in the present study indicated how many learners and teachers participated in the course, with more nodes indicating more participants. Furthermore, we investigated the network diameter, a structural feature of social networks, which is a common metric for measuring network size in SNA [30]. The network diameter refers to the longest path between any two nodes in the network. There has been evidence that a larger network diameter leads to greater spread of behavior [31]. Likewise, Gašević et al. found that larger networks were more likely to spread innovative ideas about educational technology when analyzing MOOC-related research citations [32]. Therefore, we employed node count and network diameter to measure the network’s spatial size and further explore the expansion characteristic of online courses. Brief introduction of these indicators can be summarized in Table 2.

**Table 2:** Introduction of indicators for Question (1).

Indicator	Definition	Study
**Node count**	The number of learners and teachers who have participated in the course.	Cole et al. [15]; Jan [29]
**Network diameter**	The longest path between any two nodes in the network.	Serrat O [30]; Gašević et al. [32]

To address Question (2), a list of interactive analysis metrics in SNA were introduced to scrutinize learners’ interaction characteristics in online learning during and after the pandemic, as shown below:

(1) The average degree reflects the density of the network by calculating the average number of connections for each node. As Rong and Xu suggested, the average degree of a network indicates how active its participants are [33]. According to Hu, a higher average degree implies that more students are interacting directly with each other in a learning context [34]. The present study inherited the concept of the average degree from these previous studies: the higher the average degree, the more frequent the interaction between individuals in the network.
(2) Essentially, a weighted average degree in a network is calculated by multiplying each degree by its respective weight, and then taking the average. Bydžovská took the strength of the relationship into account when determining the weighted average degree [35]. By calculating friendship’s weighted value, Maroulis assessed peer achievement within a small-school reform [36]. Accordingly, we considered the number of interactions as the weight of the degree, with a higher average degree indicating more active interaction among learners.
(3) Network density is the ratio between actual connections and potential connections in a network. The more connections group members have with each other, the higher the network density. In SNA, network density is similar to group cohesion, i.e., a network of more strong relationships is more cohesive [37]. Network density also reflects how much all members are connected together [38]. Therefore, we adopted network density to indicate the closeness among network members. Higher network density indicates more frequent interaction and closer communication among students.
(4) Clustering coefficient describes local network attributes and indicates that two nodes in the network could be connected through adjacent nodes. The clustering coefficient measures users’ tendency to gather (cluster) with others in the network: the higher the clustering coefficient, the more frequently users communicate with other group members. We regarded this indicator as a reflection of the cohesiveness of the group [39].
(5) In a network, the average path length is the average number of steps along the shortest paths between any two nodes. Oliveres has observed that when an average path length is small, the route from one node to another is shorter when graphed [40]. This is especially true in educational settings where students tend to become closer friends. So we consider that the smaller the average path length, the greater the possibility of interaction between individuals in the network.
(6) A network with a large number of nodes, but whose average path length is surprisingly small, is known as the small-world effect [41]. A higher clustering coefficient and shorter average path length are important indicators of a small-world network: a shorter average path length enables the network to spread information faster and more accurately; a higher clustering coefficient can promote frequent knowledge exchange within the group while boosting the timeliness and accuracy of knowledge dissemination [42]. Brief introduction of these indicators can be summarized in Table 3.

**Table 3:** Introduction of indicators for Question (2).

Indicators	Definition	Study
**Average degree**	The density of the network by calculating the average number of connections for each node.	Rong & Xu [33]; Hu [34]
**Weighted average degree**	Calculating the average degree based on the network’s different weights.	Bydžovská [35]; Maroulis [36]
**Network density**	The ratio between actual connections and potential connections in a network.	Wise S [37]; Potts BB [38]
**Clustering coefficient**	Two nodes in the network could be connected through adjacent nodes.	Grunspan DZ et al. [39]
**Average path length**	The average number of steps along the shortest paths between any two nodes.	Oliveres [40]
**Small-world effect**	A network with a large number of nodes, but whose average path length is surprisingly small.	Travers J et al. [41]; Watts DJ et al. [42]

To analyze Question 3, we used the concept of closeness centrality, which determines how close a vertex is to others in the network. As Opsahl et al. explained, closeness centrality reveals how closely actors are coupled with their entire social network [43]. In order to analyze social network-based engineering education, Putnik et al. examined closeness centrality and found that it was significantly correlated with grades [38]. We used closeness centrality to measure the position of an individual in the network. Brief introduction of these indicators can be summarized in Table 4.

**Table 4:** Introduction of indicators for Question (3).

Indicators	Definition	Study
**closeness centrality**	Measure the position of an individual in the network.	Opsahl [44]; Putnik [45]

### 3.4 Ethics statement

This study was approved by the Academic Committee Office (ACO) of South China Normal University (http://fzghb.scnu.edu.cn/), Guangzhou, China. Research data were collected from the open platform and analyzed anonymously. There are thus no privacy issues involved in this study.

## 4. Results

### 4.1 COVID-19’s role in promoting the scale of online courses was not as important as expected

**Fig 2:**
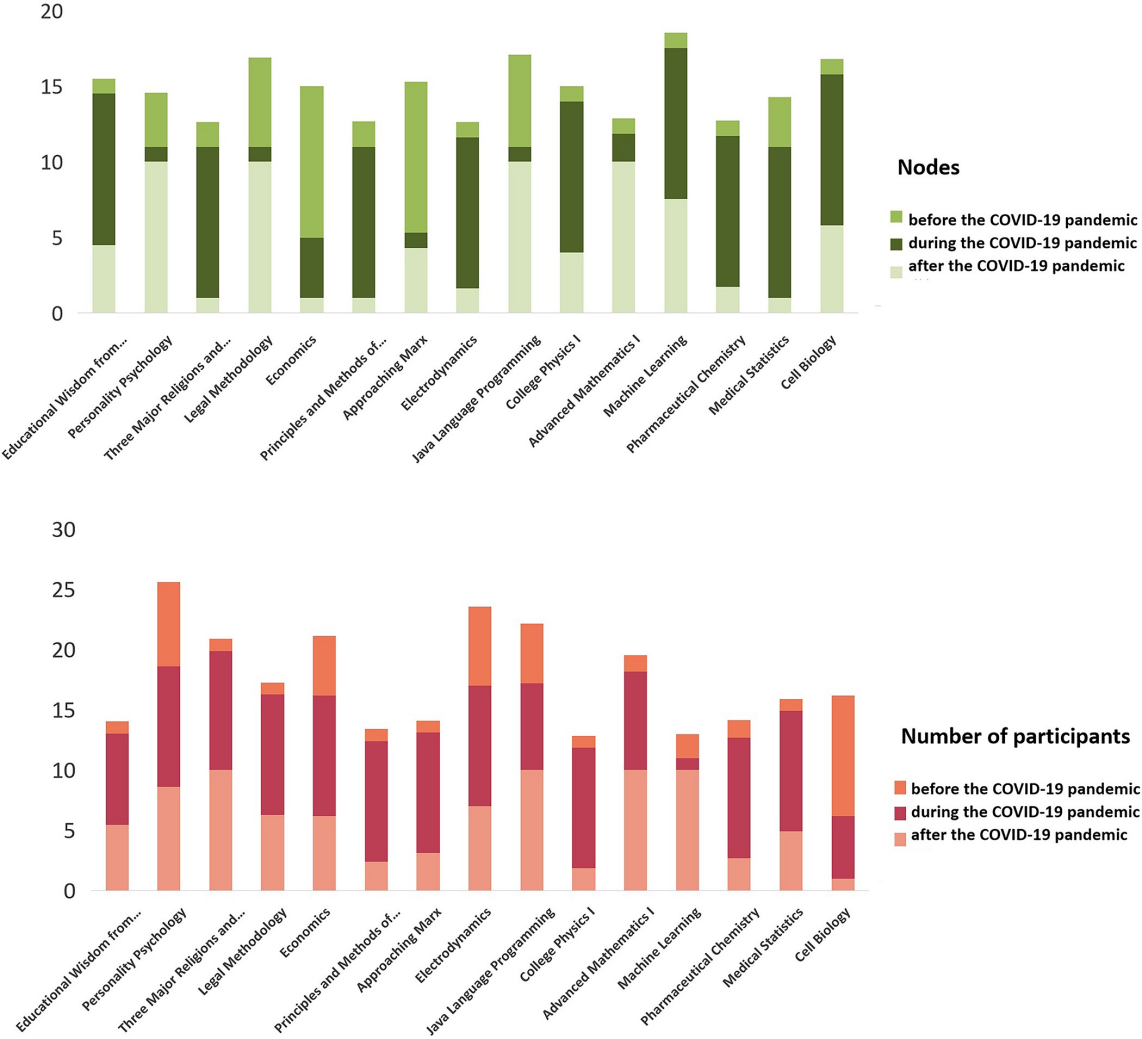
Participants and nodes in course network.

As shown in Fig 2, the number of course participants and nodes are closely correlated with the pandemic’s trajectory. Because the number of participants in each course varied widely, we normalized the number of participants and nodes to more conveniently visualize course trends. Fig 2 depicts changes in the chosen courses’ number of participants and nodes before the pandemic (Phase II), during the pandemic (Phase III), and after the pandemic (Phase IV). The number of participants in most courses during the pandemic exceeded those before and after the pandemic. But the number of people who participate in interaction in some courses did not increase.

In order to better analyze the trend of interaction scale in online courses before, during, and after the pandemic, the selected courses were categorized according to their scale change. When the number of participants increased (decreased) beyond 20% (statistical experience) and the diameter also increased (decreased), the course scale was determined to have increased (decreased); otherwise, no significant change was identified in the course’s interaction scale. Courses were subsequently divided into three categories: increased interaction scale, decreased interaction scale, and no significant change. Results appear in Table 5.

**Table 5:** Courses’ scale change.

Period	Course scale	Course coding (Social sciences)	Course coding (Natural sciences)
**Before COVID-19 pandemic–During COVID-19 pandemic**	Increase	SS5; SS6	NS1; NS3; NS8
	Decrease	-	NS2
	No significant change	SS1; SS2; SS3; SS4; SS7	NS4; NS5; NS6; NS7
**During COVID-19 pandemic–After COVID-19 pandemic**	Decrease	SS1; SS2; SS3; SS6; SS7	NS2; NS3; NS7; NS8
	No significant change	SS4; SS5	NS1; NS4; NS5; NS6

From before the pandemic until it broke out, the interaction scale of five courses increased, accounting for 33.3% of the full sample; one course’s interaction scale declined, accounting for 6.7%. The interaction scale of nine courses decreased, accounting for 60%. The pandemic’s role in promoting online courses thus was not as important as anticipated, and most courses’ interaction scale did not change significantly throughout.

No courses displayed growing interaction scale after the pandemic: the interaction scale of nine courses fell, accounting for 60%; and the interaction scale of six courses did not shift significantly, accounting for 40%. Courses with an increased scale of interaction during the pandemic did not maintain an upward trend. On the contrary, the improvement in the pandemic caused learners’ enthusiasm for online learning to wane. We next analyzed several interaction metrics to further explore course interaction during different pandemic periods.

### 4.2 Characteristics of online learning interaction amid COVID-19

#### 4.2.1 During the COVID-19 pandemic, online learning interaction in some courses became more active

**Fig 3:**
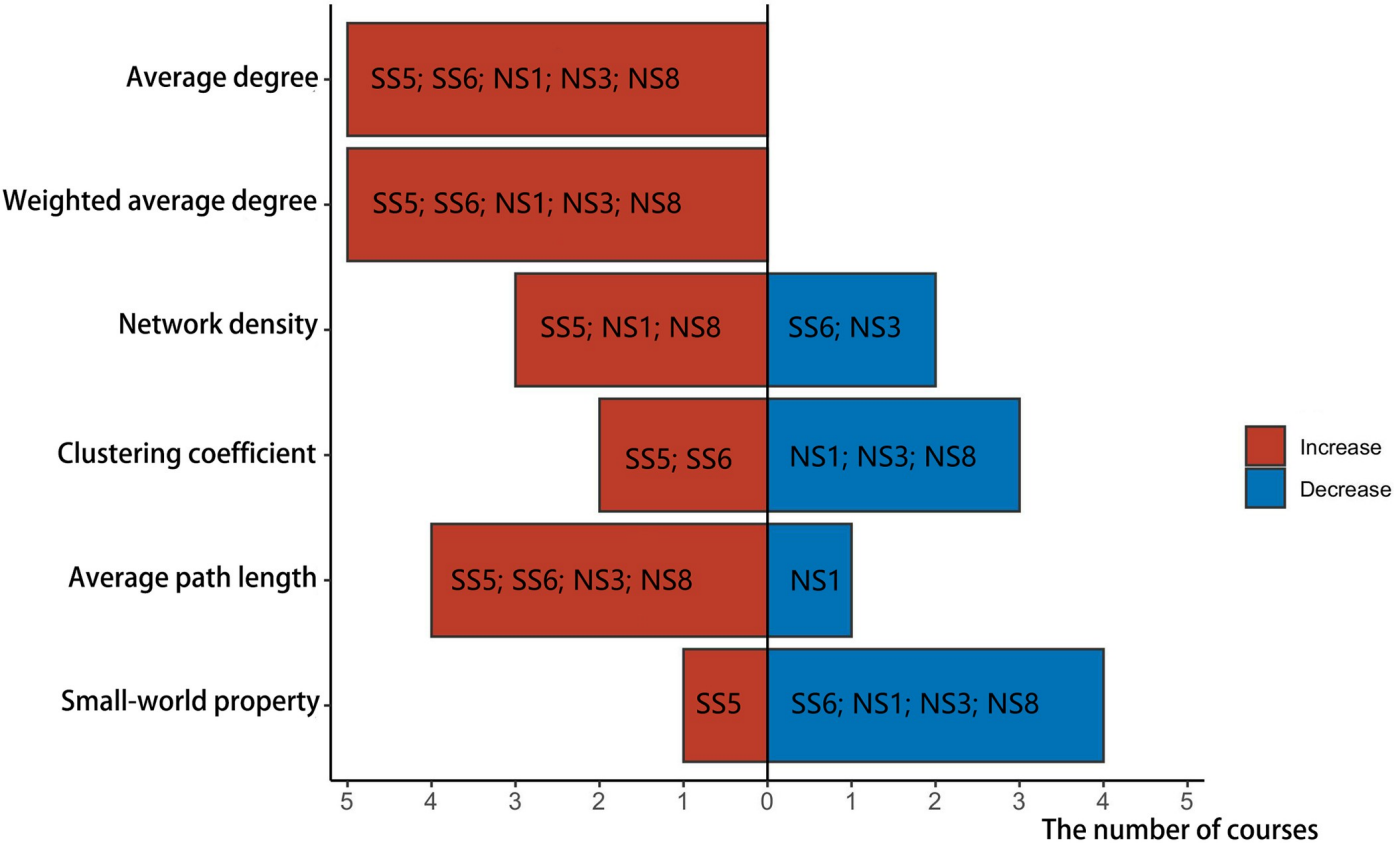
Indicator changes in courses with increased interaction scale from before the COVID-19 outbreak until the outbreak.

Changes in course indicators with the growing interaction scale during the pandemic are presented in Fig 3, including SS5, SS6, NS1, NS3, and NS8. The horizontal ordinate indicates the number of courses, with red color representing the rise of the indicator value on the vertical ordinate and blue representing the decline.

Specifically: (1) The average degree and weighted average degree of the five course networks demonstrated an upward trend. The emergence of the pandemic promoted students’ enthusiasm; learners were more active in the interactive network. (2) Fig 3 shows that 3 courses had increased network density and 2 courses had decreased. The higher the network density, the more communication within the team. Even though the pandemic accelerated the interaction scale and frequency, the tightness between learners in some courses did not improve. (3) The clustering coefficient of social science courses rose whereas the clustering coefficient and small-world property of natural science courses fell. The higher the clustering coefficient and the small-world property, the better the relationship between adjacent nodes and the higher the cohesion [39]. (4) Most courses’ average path length increased as the interaction scale increased. However, when the average path length grew, adverse effects could manifest: communication between learners might be limited to a small group without multi-directional interaction.

**Fig 4:**
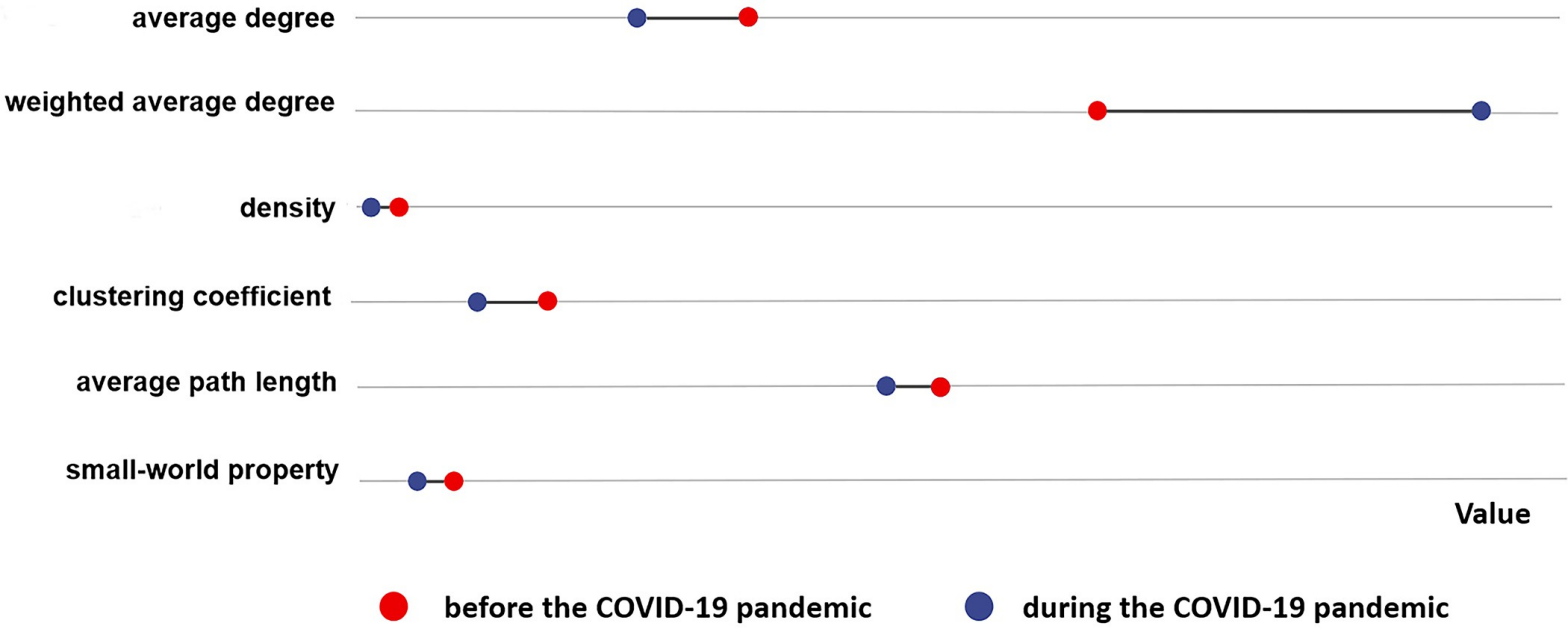
Indicator changes in courses with decreased interaction scale from before the COVID-19 pandemic until the outbreak.

When the pandemic emerged, the only declining network scale belonged to a natural science course (NS2). The change in each course index is pictured in Fig 4. The abscissa indicates the size of the value, with larger values to the right. The red dot indicates the index value before the pandemic; the blue dot indicates its value during the pandemic. If the blue dot is to the right of the red dot, then the value of the index increased; otherwise, the index value declined. Only the weighted average degree of the course network increased. The average degree, network density decreased, indicating that network members were not active and that learners’ interaction degree and communication frequency lessened. Despite reduced learner interaction, the average path length was small and the connectivity between learners was adequate.

#### 4.2.2 After the COVID-19 pandemic, the scale decreased rapidly, but most course interaction was more effective

**Fig 5:**
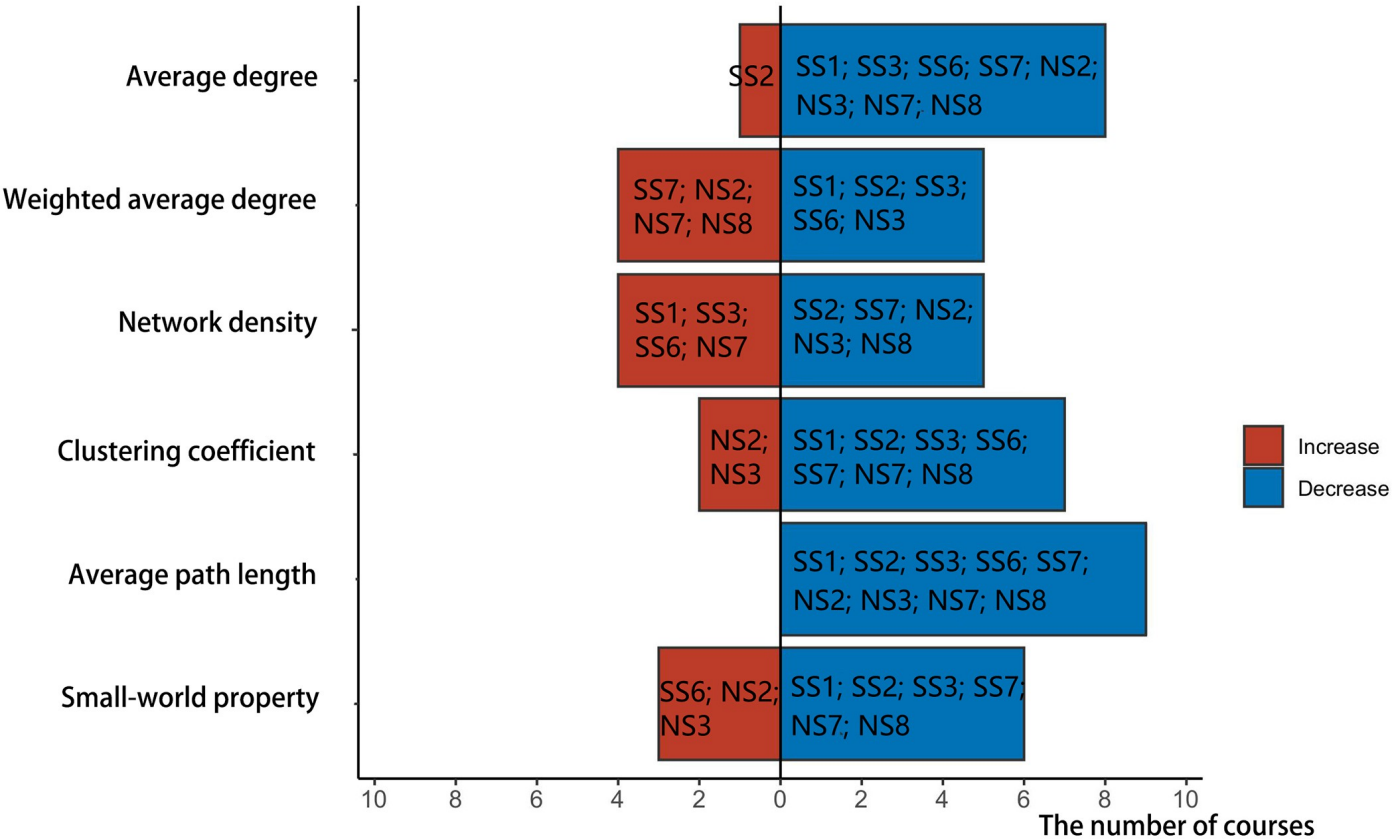
Changes in indicators of courses with decreased interaction scale from the COVID-19 outbreak to after the pandemic.

Fig 5 shows the changes in various courses’ interaction indicators after the pandemic, including SS1, SS2, SS3, SS6, SS7, NS2, NS3, NS7, and NS8.

Specifically: (1) The average degree and weighted average degree of most course networks decreased. The scope and intensity of interaction among network members declined rapidly, as did learners’ enthusiasm for communication. (2) The network density of seven courses also fell, indicating weaker connections between learners in most courses. (3) In addition, the clustering coefficient and small-world property of most course networks decreased, suggesting little possibility of small groups in the network. The scope of interaction between learners was not limited to a specific space, and the interaction objects had no significant tendencies. (4) Although the scale of course interaction became smaller in this phase, the average path length of members’ social networks shortened in nine courses. Its shorter average path length would expedite the spread of information within the network as well as communication and sharing among network members.

**Fig 6:**
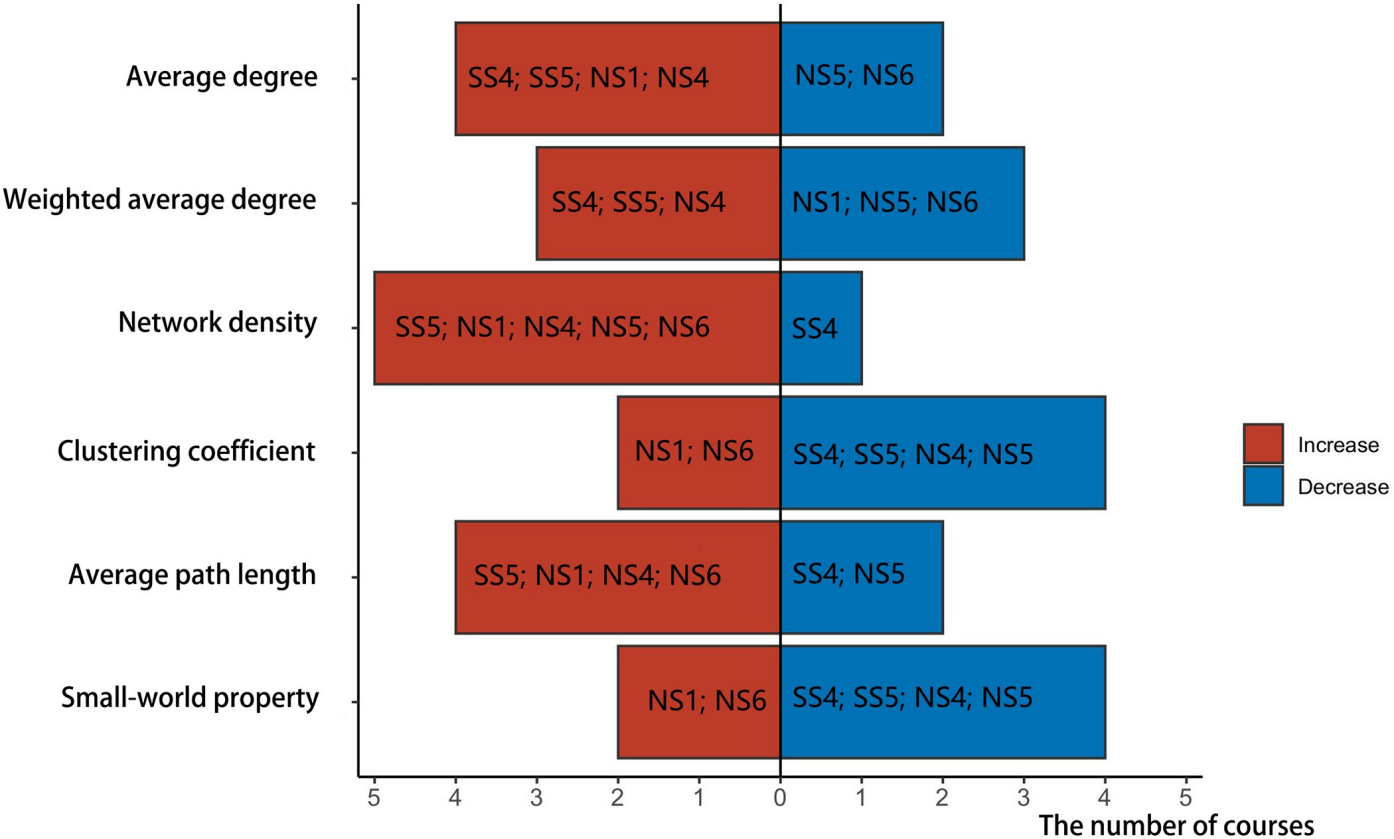
Changes in indicators of courses with no change in interaction scale from the COVID-19 outbreak until after the pandemic.

Fig 6 displays the evolution of course interaction indicators without significant changes in interaction scale after the pandemic, including SS4, SS5, NS1, NS4, NS5, and NS6.

Specifically: (1) Some course members’ social networks exhibited an increase in the average and weighted average. In these cases, even though the course network’s scale did not continue to increase, communication among network members rose and interaction became more frequent and deeper than before. (2) Network density and average path length are indicators of social network density. The greater the network density, the denser the social network; the shorter the average path length, the more concentrated the communication among network members. However, at this phase, the average path length and network density in most courses had increased. Yet the network density remained small despite having risen (Table 6). Even with more frequent learner interaction, connections remained distant and the social network was comparatively sparse.

**Table 6:** Network density.

Phase	SS4	SS5	NS1	NS4	NS5	NS6
**Phase Ⅰ**	0.05	0.033	0.043	0.003	0.048	0.024
**Phase Ⅱ**	0.077	0.031	0.038	0.002	0.04	0.043
**Phase Ⅲ**	0.5	0.033	0.004	0.003	0.039	0.013
**Phase Ⅳ**	0.107	0.038	0.098	0.004	0.111	0.041
**Phase Ⅴ**		0.083	0.083			0.058

In summary, the scale of interaction did not change significantly overall. Nonetheless, some course members’ frequency and extent of interaction increased, and the relationships between network members became closer as well. In the study, we found it interesting that the interaction scale of Economics (a social science course) course and Electrodynamics (a natural science course) course expanded rapidly during the pandemic and retained their interaction scale thereafter. We next assessed these two courses to determine whether their level of interaction persisted after the pandemic.

### 4.3 Analyses of natural science courses and social science courses

#### 4.3.1 Analyses of the interaction characteristics of Economics and Electrodynamics

Economics and Electrodynamics are social science courses and natural science courses, respectively. Members’ interaction within these courses was similar: the interaction scale increased significantly when COVID-19 broke out (Phase Ⅲ), and no significant changes emerged after the pandemic (Phase Ⅴ). We hence focused on course interaction long after the outbreak (Phase V) and compared changes across multiple indicators, as listed in Table 7.

**Table 7:** Comparison of various indicators for Economics and Electrodynamics.

Course	Phase	Number of participants	Diameter	Average degree	Weighted average	Network density	Clustering coefficient	Small-world property	Average path length
**Economics (SS5)**	Ⅲ	12382	4	0.893	1.357	0.033	0.057	0.035	1.615
	Ⅳ	8891	6	1.1	2.133	0.038	0.017	0.007	2.321
	Ⅴ	6160	1	1	2.308	0.083	0.104	0.104	1
**Electrodynamics (NS1)**	Ⅲ	4685	5	1.142	5.301	0.004	0.041	0.023	1.753
	Ⅳ	3722	2	1.667	4.444	0.098	0.2	0.111	1.805
	Ⅴ	2170	3	1	2.462	0.083	0.01	0.006	1.812

As the pandemic continued to improve, the number of participants and the diameter long after the outbreak (Phase V) each declined for Economics compared with after the pandemic (Phase IV). The interaction scale decreased, but the interaction between learners was much deeper. Specifically: (1) The weighted average degree, network density, clustering coefficient, and small-world property each reflected upward trends. The pandemic therefore exerted a strong impact on this course. Interaction was well maintained even after the pandemic. The smaller network scale promoted members’ interaction and communication. (2) Compared with after the pandemic (Phase IV), members’ network density increased significantly, showing that relationships between learners were closer and that cohesion was improving. (3) At the same time, as the clustering coefficient and small-world property grew, network members demonstrated strong small-group characteristics: the communication between them was deepening and their enthusiasm for interaction was higher. (4) Long after the COVID-19 outbreak (Phase V), the average path length was reduced compared with previous terms, knowledge flowed more quickly among network members, and the degree of interaction gradually deepened.

The average degree, weighted average degree, network density, clustering coefficient, and small-world property of Electrodynamics all decreased long after the COVID-19 outbreak (Phase V) and were lower than during the outbreak (Phase Ⅲ). The level of learner interaction therefore gradually declined long after the outbreak (Phase V), and connections between learners were no longer active. Although the pandemic increased course members’ extent of interaction, this rise was merely temporary: students’ enthusiasm for learning waned rapidly and their interaction decreased after the pandemic (Phase IV). To further analyze the interaction characteristics of course members in Economics and Electrodynamics, we evaluated the closeness centrality of their social networks, as shown in section 4.3.2.

#### 4.3.2 Analysis of the closeness centrality of Economics and Electrodynamics

**Fig 7:**
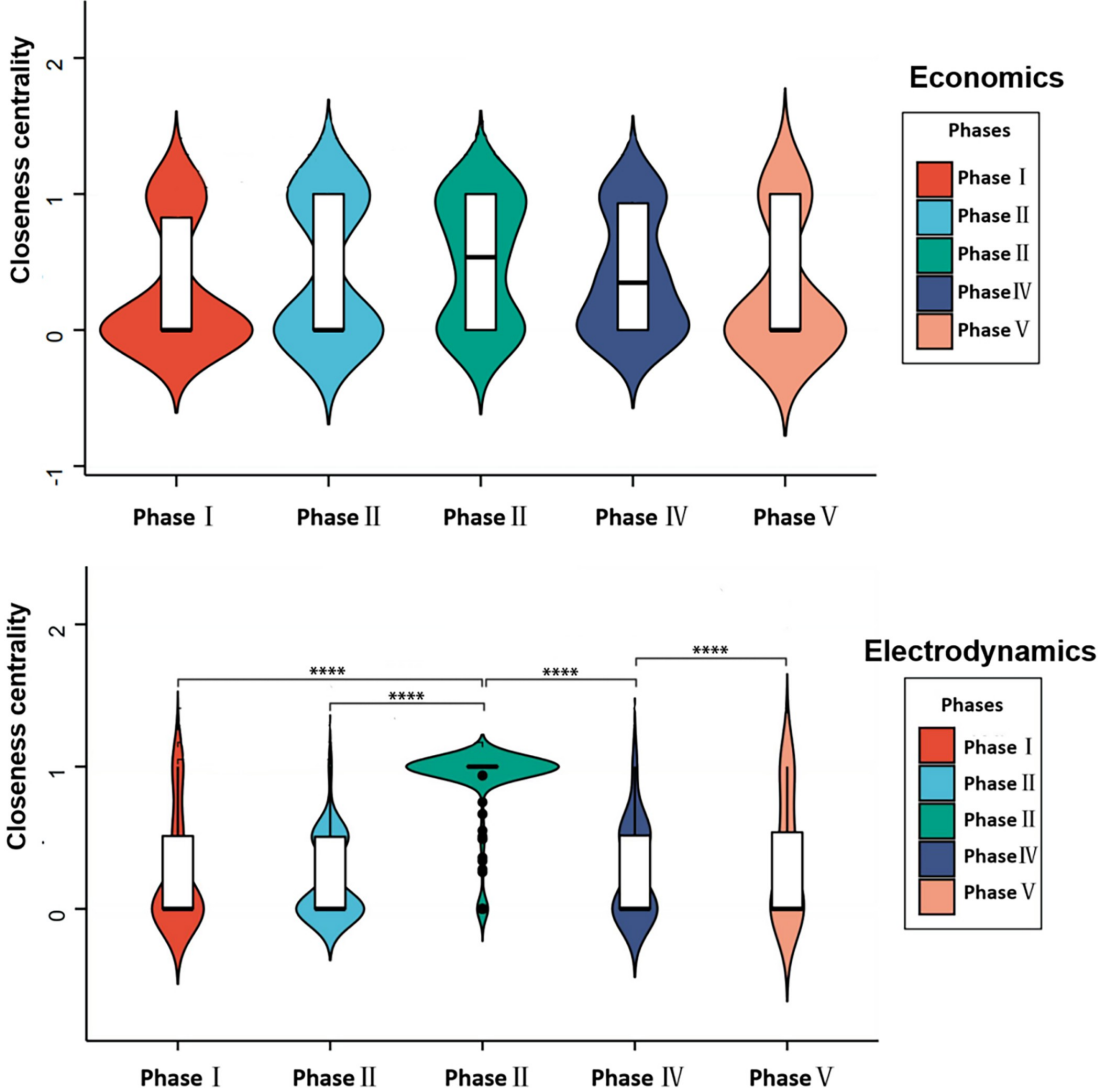
Closeness centrality of Economics and Electrodynamics. (Note: "****" indicates the significant distinction in closeness centrality between the two periods, otherwise no significant distinction).

The change in the closeness centrality of social networks in Economics was small, and no sharp upward trend appeared during the pandemic outbreak, as shown in Fig 7. The emergence of COVID-19 apparently fostered learners’ interaction in Economics albeit without a significant impact. The closeness centrality changed in Electrodynamics varied from that of Economics: upon the COVID-19 outbreak, closeness centrality was significantly different from other semesters. Communication between learners was closer and interaction was more effective. Electrodynamics course members’ social network proximity decreased rapidly after the pandemic. Learners’ communication lessened. In general, Economics course showed better interaction before the outbreak and was less affected by the pandemic; Electrodynamics course was more affected by the pandemic and showed different interaction characteristics at different periods of the pandemic.

## 5. Discussion

We referred to discussion forums from several courses on the icourse.163 MOOC platform to compare online learning before, during, and after the COVID-19 pandemic via SNA and to delineate the pandemic’s effects on online courses. Only 33.3% of courses in our sample increased in terms of interaction during the pandemic; the scale of interaction did not rise in any courses thereafter. When the courses scale rose, the scope and frequency of interaction showed upward trends during the pandemic; and the clustering coefficient of natural science courses and social science courses differed: the coefficient for social science courses tended to rise whereas that for natural science courses generally declined. When the pandemic broke out, the interaction scale of a single natural science course decreased along with its interaction scope and frequency. The amount of interaction in most courses shrank rapidly during the pandemic and network members were not as active as they had been before. However, after the pandemic, some courses saw declining interaction but greater communication between members; interaction also became more frequent and deeper than before.

### 5.1 During the COVID-19 pandemic, the scale of interaction increased in only a few courses

The pandemic outbreak led to a rapid increase in the number of participants in most courses; however, the change in network scale was not significant. The scale of online interaction expanded swiftly in only a few courses; in others, the scale either did not change significantly or displayed a downward trend. After the pandemic, the interaction scale in most courses decreased quickly; the same pattern applied to communication between network members. Learners’ enthusiasm for online interaction reduced as the circumstances of the pandemic improved—potentially because, during the pandemic, China’s Ministry of Education declared “School’s Out, But Class’s On” policy. Major colleges and universities were encouraged to use the Internet and informational resources to provide learning support, hence the sudden increase in the number of participants and interaction in online courses [46]. After the pandemic, students’ enthusiasm for online learning gradually weakened, presumably due to easing of the pandemic [47]. More activities also transitioned from online to offline, which tempered learners’ online discussion. Research has shown that long-term online learning can even bore students [48].

Most courses’ interaction scale decreased significantly after the pandemic. First, teachers and students occupied separate spaces during the outbreak, had few opportunities for mutual cooperation and friendship, and lacked a sense of belonging [49]. Students’ enthusiasm for learning dissipated over time [50]. Second, some teachers were especially concerned about adapting in-person instructional materials for digital platforms; their pedagogical methods were ineffective, and they did not provide learning activities germane to student interaction [51]. Third, although teachers and students in remote areas were actively engaged in online learning, some students could not continue to participate in distance learning due to inadequate technology later in the outbreak [52].

### 5.2 Characteristics of online learning interaction during and after the COVID-19 pandemic

#### 5.2.1 During the COVID-19 pandemic, online interaction in most courses did not change significantly

The interaction scale of only a few courses increased during the pandemic. The interaction scope and frequency of these courses climbed as well. Yet even as the degree of network interaction rose, course network density did not expand in all cases. The pandemic sparked a surge in the number of online learners and a rapid increase in network scale, but students found it difficult to interact with all learners. Yau pointed out that a greater network scale did not enrich the range of interaction between individuals; rather, the number of individuals who could interact directly was limited [53]. The internet facilitates interpersonal communication. However, not everyone has the time or ability to establish close ties with others [54].

In addition, social science courses and natural science courses in our sample revealed disparate trends in this regard: the clustering coefficient of social science courses increased and that of natural science courses decreased. Social science courses usually employ learning approaches distinct from those in natural science courses [55]. Social science courses emphasize critical and innovative thinking along with personal expression [56]. Natural science courses focus on practical skills, methods, and principles [57]. Therefore, the content of social science courses can spur large-scale discussion among learners. Some course evaluations indicated that the course content design was suboptimal as well: teachers paid close attention to knowledge transmission and much less to piquing students’ interest in learning. In addition, the thread topics that teachers posted were scarcely diversified and teachers’ questions lacked openness. These attributes could not spark active discussion among learners.

#### 5.2.2 Online learning interaction declined after the COVID-19 pandemic

Most courses’ interaction scale and intensity decreased rapidly after the pandemic, but some did not change. Courses with a larger network scale did not continue to expand after the outbreak, and students’ enthusiasm for learning paled. The pandemic’s reduced severity also influenced the number of participants in online courses. Meanwhile, restored school order moved many learning activities from virtual to in-person spaces. Face-to-face learning has gradually replaced online learning, resulting in lower enrollment and less interaction in online courses. Prolonged online courses could have also led students to feel lonely and to lack a sense of belonging [58].

The scale of interaction in some courses did not change substantially after the pandemic yet learners’ connections became tighter. We hence recommend that teachers seize pandemic-related opportunities to design suitable activities. Additionally, instructors should promote student-teacher and student-student interaction, encourage students to actively participate online, and generally intensify the impact of online learning.

### 5.3 What are the characteristics of interaction in social science courses and natural science courses?

The level of interaction in Economics (a social science course) was significantly higher than that in Electrodynamics (a natural science course), and the small-world property in Economics increased as well. To boost online courses’ learning-related impacts, teachers can divide groups of learners based on the clustering coefficient and the average path length. Small groups of students may benefit teachers in several ways: to participate actively in activities intended to expand students’ knowledge, and to serve as key actors in these small groups. Cultivating students’ keenness to participate in class activities and self-management can also help teachers guide learner interaction and foster deep knowledge construction.

As evidenced by comments posted in the Electrodynamics course, we observed less interaction between students. Teachers also rarely urged students to contribute to conversations. These trends may have arisen because teachers and students were in different spaces. Teachers might have struggled to discern students’ interaction status. Teachers could also have failed to intervene in time, to design online learning activities that piqued learners’ interest, and to employ sound interactive theme planning and guidance. Teachers are often active in traditional classroom settings. Their roles are comparatively weakened online, such that they possess less control over instruction [59]. Online instruction also requires a stronger hand in learning: teachers should play a leading role in regulating network members’ interactive communication [60]. Teachers can guide learners to participate, help learners establish social networks, and heighten students’ interest in learning [61]. Teachers should attend to core members in online learning while also considering edge members; by doing so, all network members can be driven to share their knowledge and become more engaged. Finally, teachers and assistant teachers should help learners develop knowledge, exchange topic-related ideas, pose relevant questions during course discussions, and craft activities that enable learners to interact online [62]. These tactics can improve the effectiveness of online learning.

As described, network members displayed distinct interaction behavior in Economics and Electrodynamics courses. First, these courses varied in their difficulty: the social science course seemed easier to understand and focused on divergent thinking. Learners were often willing to express their views in comments and to ponder others’ perspectives [63]. The natural science course seemed more demanding and was oriented around logical thinking and skills [64]. Second, courses’ content differed. In general, social science courses favor the acquisition of declarative knowledge and creative knowledge compared with natural science courses. Social science courses also entertain open questions [65]. Natural science courses revolve around principle knowledge, strategic knowledge, and transfer knowledge [66]. Problems in these courses are normally more complicated than those in social science courses. Third, the indicators affecting students’ attitudes toward learning were unique. Guo et al. discovered that “teacher feedback” most strongly influenced students’ attitudes towards learning social science courses but had less impact on students in natural science courses [67]. Therefore, learners in social science courses likely expect more feedback from teachers and greater interaction with others.

## 6. Conclusion and future work

Our findings show that the network interaction scale of some online courses expanded during the COVID-19 pandemic. The network scale of most courses did not change significantly, demonstrating that the pandemic did not notably alter the scale of course interaction. Online learning interaction among course network members whose interaction scale increased also became more frequent during the pandemic. Once the outbreak was under control, although the scale of interaction declined, the level and scope of some courses’ interactive networks continued to rise; interaction was thus particularly effective in these cases. Overall, the pandemic appeared to have a relatively positive impact on online learning interaction. We considered a pair of courses in detail and found that Economics (a social science course) fared much better than Electrodynamics (a natural science course) in classroom interaction; learners were more willing to partake in-class activities, perhaps due to these courses’ unique characteristics. Brint et al. also came to similar conclusions [57].

This study was intended to be rigorous. Even so, several constraints can be addressed in future work. The first limitation involves our sample: we focused on a select set of courses hosted on China’s icourse.163 MOOC platform. Future studies should involve an expansive collection of courses to provide a more holistic understanding of how the pandemic has influenced online interaction. Second, we only explored the interactive relationship between learners and did not analyze interactive content. More in-depth content analysis should be carried out in subsequent research. All in all, the emergence of COVID-19 has provided a new path for online learning and has reshaped the distance learning landscape. To cope with associated challenges, educational practitioners will need to continue innovating in online instructional design, strengthen related pedagogy, optimize online learning conditions, and bolster teachers’ and students’ competence in online learning.
